# Identification of a Novel *TECTA* Mutation in a Chinese DFNA8/12 Family with Prelingual Progressive Sensorineural Hearing Impairment

**DOI:** 10.1371/journal.pone.0070134

**Published:** 2013-07-31

**Authors:** Zhengyue Li, Yilian Guo, Yu Lu, Jianzhong Li, Zhanguo Jin, Hongbo Li, Yanping Lu, Pu Dai, Dongyi Han, Jing Cheng, Huijun Yuan

**Affiliations:** 1 Institute of Otolaryngology, Chinese PLA General Hospital, Beijing, China; 2 Department of Otolaryngology, Zhongshan Traditional Chinese Medicine Hospital of Guangdong Province, Zhongshan, GuangDong Province, China; 3 Department of Otolaryngology,FuZhou General Hospital of NanJing Command PLA, FuZhou, Fujian Province, China; 4 Department of Obstetrics and Gynecology, Chinese PLA General Hospital, Beijing, China; University of Sydney, Australia

## Abstract

Tectorial membrane, an extracellular matrix of the cochlea, plays a crucial role in the transmission of sound to the sensory hair cells. Alpha-tectorin is the most important noncollagenous component of the tectorial membrane and the otolith membrane in the maculae of the vestibular system. Defects in *TECTA*, the gene encodes alpha-tectorin, are cause of both dominant (DFNA8/12) and recessive (DFNB21) forms of deafness. Here, we report a three-generation Chinese family characterized by prelingual progressive sensorineural hearing impairment. We mapped the disease locus to chromosome 11q23-24 region, overlapping with the DFNA8/12 locus. Sequencing of candidate gene *TECTA* revealed a heterozygous c.5945C>A substitution in exon 19, causing amino acid substitution of Ala to Asp at a conservative position 1982. The A1982D substitution is consistent with hearing loss in this Chinese family and has not been found in 200 random control chromosomes. To our knowledge, this is the first *TECTA* mutation identified in Chinese population. Our data provides additional molecular and clinical information for establishing a better genotype–phenotype understanding of DFNA8/12.

## Introduction

The tectorial membrane of the inner ear is a ribbon-like strip of extracellular matrix that spirals along the entire length of the cochlea. It attaches along its medial side to the surface of the spiral limbus, stretches across the spiral sulcus, and lies over the surface of the organ of Corti where it affixes to the tips of the sensory hair bundles of the outer hair cells [1]. Upon sound stimulation, the relative displacement of the tectorial membrane with regards to the hair cells provokes a deflection of their stererociliary bundles, thereby leading to the opening of their mechanotransduction channel [2]. The deflection of stereocilium bundles is enabled by the anisotropic properties of the tectorial membrane. Tectorial membrane is composed of several genetically distinct types of collagen, collagen Types II, V, IX and XI and three non-collagenous glycoproteins, α-tectorin (Tecta), β-tectorin (Tectb) and otogelin. Tecta, Tectb and otogelin are proteins that are only expressed at high levels in the inner ear and they account for ∼50% of the protein present in the tectorial membrane [3],[4],[5],[6]. The cDNA sequences for otogelin and Tecta predict large, modular glycoproteins (313 and 239 kDa respectively), whilst the sequence for Tectb encodes a much smaller protein (36 kDa). Alpha-tectorin is encoded by *TECTA* gene which has 23 exons. It consists of a polypeptide of 2,155 amino acids and is composed of 3 distinct modules: entactin G1 domain, zonadhesin domain (with von Willebrand factor type D repeat), and zona pellucida domain. There 3 polypeptides are crosslinked to each other by disulfide bridges and interact with β-tectorin to form the noncollagenous matrix of the tectorial membrane [2]. An alteration of α-tectorin is likely to disrupt the structure of this matrix and, in consequence, to lead to inefficient transmission of sound. Defects in *TECTA* are cause of both dominant (DFNA8/12, OMIM 601543) and recessive (DFNB21, OMIM 603629) forms of deafness, and absence of *TECTA* gene is responsible for Jacobsen syndrome (OMIM 147791), a contiguous gene deletion syndrome involving terminal chromosome 11q [7],[8],[9],[10]. In this article, we report a Chinese DFNA8/12 family with prelingual progressive sensorineural hearing impairment. We mapped a disease locus at chromosome 11q23-24 and determined that a novel heterozygous missense mutation in exon 19 of *TECTA* is likely responsible for the phenotype in this family.

## Materials and Methods

### Clinical Evaluation

A Chinese family, GD-O031, with congenital hearing loss, was ascertained from Department of Otolaryngology, Zhongshan Traditional Chinese Medicine Hospital of Guangdong Province in mainland China. This study was approved by the Ethnic Committee of Chinese PLA General Hospital. Written informed consent was obtained from the adult participants and the guardians on the behalf of the children prior to their participation in the study. The medical history was obtained by use of a questionnaire regarding the following aspects: subjective degree of hearing loss, age at onset, evolution, symmetry of the hearing impairment, hearing aids, presence of tinnitus, pressure in the ears or vertigo, medication, noise exposure, pathologic changes in the ear, and other relevant clinical manifestations. Otoscopy, physical examination, and pure tone audiometry (at frequencies from 250 to 8,000 Hz) were performed. The degree of hearing loss was defined according to pure-tone averages (PTA), which were based on the three frequencies of 500, 1,000 and 2,000 Hz. Immittance testing was applied to evaluate middle ear pressures, ear canal volumes, and tympanic membrane mobility.

### Linkage Analysis of Known DFNA Loci

Blood samples (3 ml) were drawn from 18 participants for DNA extraction and were used for genetic analysis. Genomic DNA was extracted using the Genomic DNA isolation kit (HuaShun, Shanghai). Fluorescently labeled microsatellite markers for the exclusion of 24 known DFNA loci (DFNA 1, 2a, 2b, 3, 4, 5, 6/14/38, 8/12, 9, 10, 11, 13, 15, 17, 20, 22, 25, 28, 36, 39, 44, 48, 50, 51) were taken from the hereditary hearing loss homepage (http://webhost.ua.ac.be/hhh) and the Marshfield map (http://research.marshfieldclinic.org/genetics). The microsatellite markers were amplified by the PCR on a Gene AmpPCR system 9700 (Perkin Elmer, USA) and were analyzed on ABI 3730 Genetic Analyzer (AppliedBiosystems, USA). The alleles were assigned by using Genescan and Genotyper Software (AppliedBiosystems, USA). Linkage analysis was performed by using the LINKAGE 5.1 software package. Two-point LOD score between the deafness locus and each marker was calculated under a fully penetrant autosomal dominant mode of inheritance, setting the disease allele frequency to 0.0001. The meiotic recombination frequencies were considered to be equal for males and females.

### Mutational Analysis

Direct sequencing was used for mutation screening. The primers were designed to amplify all exons and flanking intronic splicing sites of the *TECTA* gene (NM_005422). Sequences of these primers are provided in supplementary Table S1. PCR was performed according to standard conditions. Bi-directional sequencing was carried out using both the forward and reverse primers and was performed using the ABI PRISM Big Dye Terminator cycle sequencing ready reaction kit on a 3130 ABI DNA-sequencer (Applied Biosystems, USA). One hundred DNA samples from a panel of unaffected individuals from Chinese background comprised the control genomic DNA samples. ClustalW2 was applied to make the alignment of the TECTA protein from different species.

## Results and Discussion

**Figure 1:**
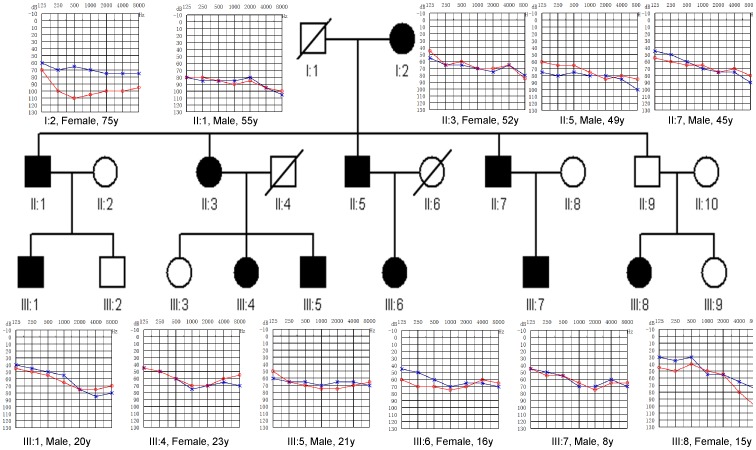
Pedigree of three-generation Chinese family GD-O031 with prelingual progressive hearing impairment and audiograms of 11 affected subjects. Affected subjects are denoted in black. Based on the audiograms of the affected subjects (red, right ear; black, left ear), the severity of hearing impairment was moderate to severe and progress slowly with increasing patient age. The hearing loss involved all frequencies.

The pedigree of family GD-O031, which spanning three generations and comprising 21 members, is consistent with an autosomal dominant inheritance pattern (DFNA). Eighteen family members including 11 patients and 7 individuals with normal hearing participated in this study. Based on the questionnaires, hearing impairment of 10 affected subjects (except individual III-8) was prelingual and symmetric. The severity of hearing impairment was moderate (8 cases) to severe (2 cases) and progressed slowly with increasing patient age. The hearing loss involved all frequencies and the audiograms of affected members have a flat contour (Figure 1). Audiologic evaluation of the family members demonstrates normal immittance testing and bone conduction values that equal the air conduction measurements, suggesting sensorineural hearing impairment. Unlike other affected family members, the age at onset of hearing impairment of individual III-8 was 8 years. This individual had a history of the use of Gentamicin (dosage uncertained) at the age of onset. The fact that his father II-9 was unaffected further suggests that III-8 was a phenocopy. Two affected members, II-1 and II-3, have the history of noise exposure. Occasional bilateral high frequency tinnitus was reported by I-2, III-4 and III-8. The vestibular symptom, vertigo, was only reported by III-8 (Table S1). Comprehensive family medical histories and clinical examination of these individuals showed no other clinical abnormalities, including diabetes, cardiovascular diseases, visual problems and neurological disorders. Computer tomography scan analysis of the proband of family GD-O031 ruled out inner ear malformations.

**Figure 2:**
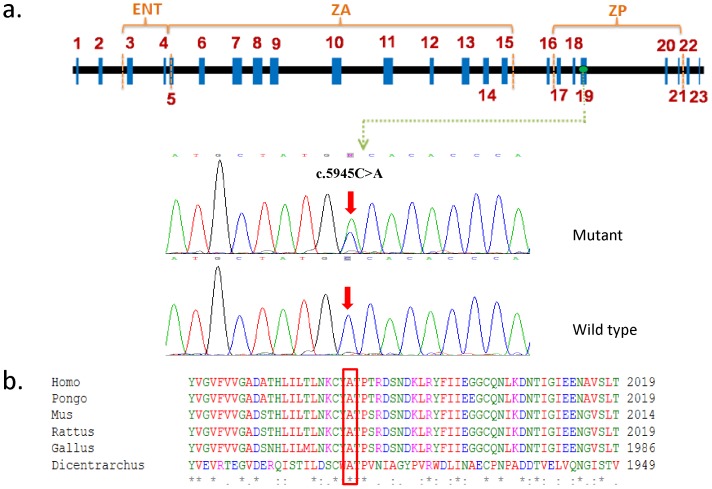
Mutation analysis of the Chinese family GD-O031. a. DNA sequence chromatograms showing heterozygous missense c.5945C>A mutation, compared to wild-type controls. The structure of TECTA depicts the position of c.5945C>A mutation in exon 19 and p.A1982D in ZP domain. b. Conservation analysis shows that the Ala residue at 1982 in TECTA is conserved across human, chimpanzee, mouse, chicken, and fish.

Eighteen members from family GD-O031 considered informative were selected for linkage analysis. We initially tested the family for linkage to 24 DFNA loci with known genes. Negative results were obtained excluding the involvement of 23 DFNA loci in family GD-O031 (data not shown). Evidence of linkage was found for marker *D11S4157* and *D11S4089* (two-point LOD score was 3.17 and 5.31 at [theta] = 0, respectively) on chromosome 11q23-24 region, overlapping with the DFNA8/12 locus. The maximum LOD score is 5.31 (*D11S4089*). The candidate gene *TECTA* is localized within this region. We sequenced all 23 coding exons of *TECTA* in two affected (I-2 and II-3) and one unaffected (II-9) family members. The sequence analysis revealed a heterozygous C-to-A transition at position 5945 in exon 19, leading to a p.A1982D substitution at the zona pellucida domain of TECTA (Figure 2a). The c.5945C>A variation is not present in the exome sequence variant server (http://evs.gs.washington.edu/EVS/). The Ala residue at 1982 in TECTA is conserved across human, chimpanzee, mouse, chicken, and fish (Figure 2b). The heterozygous nucleotide change is consistent with an autosomal dominant pattern of inheritance for hearing loss. Sequence analysis demonstrated that the c.5945C>A substitution faithfully co-segregated with hearing loss in the family and that it was absent in 200 unrelated control chromosomes of Chinese background, supporting the hypothesis that it represents a causative mutation, not a rare polymorphism. Homozygous c.2795T>C (exon9) and c.5171G>A (exon15) variations were detected in individual I-2 during the sequence analysis. But these variations did not cosegregate with hearing loss in this family.

Human tectorial membrane is formed between the 12th and 20th weeks of embryonic development. α-Tectorin is only expressed transiently during cochlear development [1]. Up to date, forty-one DFNA8/12 families and seven DFNB21 families have been reported worldwide. Based on these reports, typical affected individuals in DFNA8/12 families usually experienced prelingual and nonprogressive hearing loss between 60 and 80 dB, with a maximum at 2,000 Hz (severe range 1,000 to 6,000 kHz) and a U-shaped curve [7]. In contrast, the hearing loss in autosomal recessive families (DFNB21) is always characterized by prelingual onset and a stationary pattern. The severity of hearing loss was severe to profound (70–110 dB) and all frequencies were affected [8], [12]. In this paper, we present a detailed analysis of the genotype and phenotype of the first Chinese DFNA8/12 family based on the clinical data of 10 affected members. The prelingual-onset, progressive hearing loss phenotype segregating in this Chinese DFNA8/12 family is similar to that of reported DFNA8/12 families. However, the audiogram pattern of affected individuals was different. Typical DFNA8/12 families showed mid- or mid-high-frequency hearing impairment, while our Chinese DFNA8/12 family demonstrated moderate to severe all-frequency hearing impairment, similar to the audiological features of typical DFNB21 families. Moreover, atypical postlingual progressive high-frequency hearing impairment was previously reported in a Swedish DFNA8/12 family with a late onset around age 9 years [9]. The explanation for the different phenotypes among DFNA8/12 families lay in the localization of the mutations in different modules of the protein according to the previous studies. It was suggested that mutations localized in the zona pellucida domain of alpha-tectorin result in prelingual, nonprogressive mid-frequency hearing loss, whereas those in the zona adhesin domain result in progressive, high frequency hearing loss with onset in childhood [11]. However, recent studies suggested that the phenotypes of *TECTA* mutations detected in ZP domain can be markedly diverse (Table S2). Among 15 mutations (13 dominant and 2 recessive) identified in ZP domain, nine of them (all dominant) result in postlingual, progressive or nonprogressive hearing impairment involving high or mid frequencies, while four of them (3 dominant and 1 recessive) provoke prelingual nonprogressive mid-frequency hearing impairment. However, the phenotype of the heterozygous c.5945C>A mutation identified in this study, is more similar to the phenotype of a homozygous inactivating c.6203-6218del mutation identified in an Iranian DFNB21 family [12].

In summary, we have identified a novel missense mutation of *TECTA* in a Chinese DFNA8/12 family characterized by prelingual-onset, progressive all-frequency hearing loss. The novel *TECTA* mutation affect single amino acid conserved across species and not present in controls. Our data provides additional molecular and clinical information to understand the genotype and phenotype of *TECTA* mutations. Further molecular understanding of DFNA8/12 may allow the design of appropriate medical management and therapeutic options for this specific disorder.

## Supporting Information

Table S1 Summary of Clinical Data of Affected Individuals of Family GD-O031.
(DOCX)

Table S2 Comparison of the identified dominant *TECTA* mutations in alpha-tectorin ZP domain.
(DOCX)
