# Supplementary material for: Identification of a Novel TECTA Mutation in a Chinese DFNA8/12 Family with Prelingual Progressive Sensorineural Hearing Impairment
Source: PLoS One. 2013 Jul 31;8(7):e70134. doi: 10.1371/journal.pone.0070134 (PMC3729559; doi:10.1371/journal.pone.0070134)
Supplement: Table S1 — Summary of Clinical Data of Affected Individuals of Family GD-O031. (DOCX) [file pone.0070134.s001.docx]

Table S1. Summary of Clinical Data of Affected Individuals of Family GD-O031

| Patient | Gender | Age (years) | | Use ofAminoglycoside | Hearing TestPTA^a^ (dB) | | Audiogramshape | Tinnitus | vertigo | Noise exposure |
| --- | --- | --- | --- | --- | --- | --- | --- | --- | --- | --- |
|  |  | At Testing At Onset | |  | Left ear Right ear | |  |  |  |  |
| I-2 | Female | 75 | Prelingual | No | 71 | 104 | Flat | Yes | No | No |
| II-1 | Male | 55 | Prelingual | No | 86 | 89 | Flat | No | No | Yes |
| II-3 | Female | 52 | Prelingual | No | 69 | 66 | Flat | No | No | Yes |
| II-5 | Male | 49 | Prelingual | No | 80 | 76 | Flat | No | No | No |
| II-7 | Male | 45 | Prelingual | No | 70 | 69 | Flat-sloping | No | No | No |
| III-1 | Male | 20 | Prelingual | No | 66 | 68 | Flat-sloping | No | No | No |
| III-4 | Female | 23 | Prelingual | No | 66 | 65 | Flat | Yes | No | No |
| III-5 | Male | 21 | Prelingual | No | 66 | 73 | Flat | No | No | No |
| III-6 | Male | 16 | Prelingual | No | 65 | 69 | Flat | No | No | No |
| III-7 | Male | 8 | Prelingual | No | 64 | 65 | Flat | No | No | No |
| III-8 | Female | 19 | 8 | Yes | 51 | 56 | Sloping | Yes | Yes | No |

^a^ PTA = pure-tone average
