# Supplementary material for: Identification of a Novel TECTA Mutation in a Chinese DFNA8/12 Family with Prelingual Progressive Sensorineural Hearing Impairment
Source: PLoS One. 2013 Jul 31;8(7):e70134. doi: 10.1371/journal.pone.0070134 (PMC3729559; doi:10.1371/journal.pone.0070134)
Supplement: Table S2 — Comparison of the identified dominant TECTA mutations in alpha-tectorin ZP domain. (DOCX) [file pone.0070134.s002.docx]

Table S2. Comparison of the identified dominant *TECTA* mutations in alpha-tectorin ZP domain

| Inheritance pattern | Mutation | Location (Exon) | Age at Onset | Progression | Frequencies | Severity ofhearing loss | Familyorigin | Reference |
| --- | --- | --- | --- | --- | --- | --- | --- | --- |
| AD | c.5458C>T p.L1820F | 17 | Postlingual | Stable | Mid | Mild to severe | Belgian | Verhoeven et al., 1998^[1]^ |
| AD | c.5471G>A p.G1824D | 17 | Postlingual | Stable | Mid | Mild to severe | Belgian | Verhoeven et al., 1998^[1]^ |
| AD | c.5509T>G p.C1837G | 17 | Postlingual | Progressive | Mid | Mild to severe | Spanish | Moreno-Pelayo et al.,2001^[2]^/ Hildebrand et al., 2011^[3]^ |
| AD | c.5509T>C p.C1837R | 17 | Postlingual | Progressive | Mid | Mild to moderate | American | Meyer et al., 2007^[4]^ |
| AD | c.5597C>T p.T1866M | 18 | Postlingual | Stable/ Progressive | Mid | Mild to moderate | Korean/Spanish/ American | Sagong et al., 2010^[5]^ / Hildebrand et al.,2011^[4]^ |
| AD | c.5600A>G p.H1867R | 18 | Postlingual | Progressive | Mid | Unknown | Spanish | Hildebrand et al.,2011^[4]^ |
| AD | c.5609A>G p.Y1870C | 18 | Prelingual | Stable | Mid | Moderate to severe | Austrian | Verhoeven et al., 1998 ^[1]^ |
| AD | c.5668C>T p.R1890C | 18 | Prelingual | Stable | Mid | Mild to moderate | Dutch/Spanish/ American | Plantinga et al., 2006^[6]^ /  Hildebrand et al.,2011^[3]^ |
| AD | c.5692T>C p.C1898R | 18 | Postlingua | Unknown | Mid | Unknown | American | Hildebrand et al.,2011^[3]^ |
| AD | c.5839C>T p.R1947C | 19 | Postlingual | Unknown | Mid | Unknown | American | Hildebrand et al.,2011 ^[3]^ |
| AD | c.5945C>A p.A1982D | 19 | Prelingual | Progressive | All freq. | Moderate to severe | Chinese | This work |
| AD | c.6026T>C p.I2009T | 20 | Postlingual | Stable | High | Unknown | Spanish | Hildebrand et al.,2011 ^[3]^ |
| AD | c.6062G>A p.R2021H | 20 | Prelingual | Stable | Mid | Mild to moderate | Japanese | Iwasaki et al., 2002 ^[7]^ |

**^References^**

1. Verhoeven K, Van Laer L, Kirschhofer K, Legan PK, Hughes DC, et al. (1998) Mutations in the human alpha-tectorin gene cause autosomal dominant non-syndromic hearing impairment. Nat Genet 19: 60-62.
2. Moreno-Pelayo MA, del Castillo I, Villamar M, Romero L, Hernandez-Calvin FJ, et al. (2001) A cysteine substitution in the zona pellucida domain of alpha-tectorin results in autosomal dominant, postlingual, progressive, mid frequency hearing loss in a Spanish family. J Med Genet 38:E13.
3. Hildebrand MS, Morin M, Meyer NC, Mayo F, Modamio-Hoybjor S, et al. (2011) DFNA8/12 Caused by TECTA Mutations is the Most Identified Subtype of Nonsyndromic Autosomal Dominant Hearing Loss. Hum Mutat 32:825–834.
4. Meyer NC, Nishimura CJ, McMordie S, Smith RJ. (2007) Audioprofiling identifies TECTA and GJB2-related deafness segregating in a single extended pedigree. Clin Genet 72:130–137.
5. Sagong B, Park R, Kim YH, Lee KY, Baek JI, et al. (2010) Two novel missense mutations in the TECTA gene in Korean families with autosomal dominant nonsyndromic hearing loss. Ann Clin Lab Sci 40:380–385.
6. Plantinga RF, de Brouwer AP, Huygen PL, Kunst HP, Kremer H, et al. (2006) A novel TECTA mutation in a Dutch DFNA8/12 family confirms genotype– phenotype correlation. J Assoc Res Otolaryngol 7:173–181.
7. Iwasaki S, Harada D, Usami S, Nagura M, Takeshita T,et al (2002) Association of clinical features with mutation of TECTA in a family with autosomal dominant hearing loss. Arch Otolaryng Head Neck Surg 128: 913-917.
